# Legal Limitation of Family Limitation

**Published:** 1915-07

**Authors:** G. Henri Bogart

**Affiliations:** Paris, Ill.; San Antonio


### Legal Limitation of Family Limitation.

BY DR. G. HENRI BOGART, PARIS, ILL.

It is almost impossible to loose our clutch on the past; to turn triumphant to the glorious, glowing future.

Time was when child-bearing, as abundantly as possible, was demanded as the highest patriotism and the sunset of that era tinctures the vision of the majority who refuse to turn their faces eastward to the dawning of the day of higher quality of offspring with better opportunity. Every mechanical, chemical or electrical energy, or other storehouse of infinity that is unlocked, means more and more for the well-born, the trained and the intelligent; with more of deprivation and suffering and idleness for the ill-born, the untrained, the human machines; each mechanical advance means a corresponding cheapening of the muscled machine. With the death rate of white America at but 57 per cent of the births, the thoughtful economist wonders how we are adequately to provide for the geometric increase. Estimate for yourself what this will mean in a century. It is true that we are hearing a horrible howl about "race suicide." It is true that we have listened to woeful prophecies on the decadence of France. That nation has at least allowed the French mothers to know and practice the knowledge that shall give her the same right to exercise equal discretion in the crop of boys and girls she shall produce and rear properly that is granted her with her hens and rabbits. A proof of a theory is found in its results. France has presented to the world her matured children of love and desire, who have dumfounded us by hurling back the millions at the Marne; France, from the industry and thrift of its common folk, has been able to finance this war from the blue stocking bank; France has presented a new race with firm, optimistic fortitude and lofty national courage which tells that France has been breeding men and women of quality, rather than of mere mass quantity.

Here in America we are justly and anxiously alarmed at the swelling tide of degeneracy and feeble-mindedness that rises higher and higher each day of each year. There is a law on the Federal statute books that directly accounts for this; a law that imperils every honest, sympathetic physician.

Section 10,415 of the Federal Statutes, being Section 245 of the Criminal Code, reads:

> "Whoever shall bring or cause to be brought into the United States or any place subject to the jurisdiction thereof, or shall deposit or cause to be deposited with any express company or other common carrier, for carriage from one State, Territory or District of the United States, or place non-contiguous to but subject to the jurisdiction thereof, to any other State, Territory or District thereof, any book, paper, letter, writing, print or other matter, or any drug, medicine, article or thing, designed, intended or adapted for preventing conception, or any written, or printed card, letter, circular, book, pamphlet advertisement or notice of any kind giving direction, directly or indirectly where, how or of whom or by what means any of the hereinbefore mentioned articles, matters or things may be obtained or made; or whoever shall knowingly take or cause to be taken from any express company, or other common carrier, any matter, article or thing, the depositing of which for carriage is herein made unlawful, shall be fined not more than $5000 or imprisoned not more than five years, or both."

Doctor, do you note that there is no extenuating circumstance, no medical necessity, to soften the rigors of this most infamous law?

For instance, you have a patient whose pelvis is so distorted that her child must be dismembered and taken away; it is absolutely certain that she can never mother a living child; she has her absolute right to sexual relations, as has her husband, yet this law makes no provision for your procuring appliances, or writing prescriptions. And, no matter how desirous you might be to help her, you will find no reliable literature, for, even if published, it would be barred from transportation by the inflexible, intolerance of this law.

Not only so, but through decoy letters, men of prominence and of the highest honor have been driven into bankruptcy and the convict's cell.

I have been the target of these decoys, the most pathetic being that of a woman of culture whose husband had been hopelessly crippled in an accident, leaving her to provide for him and her little ones. She was a womanly woman, and she loved her husband. The coming of another child would mean an enforced stoppage of her ability to work, and an added burden, with corresponding lessening of opportunity for the children born before the evil days had come upon her. Could I tell her how to avoid further pregnancy? I could but I did not, because I dared not. I could have told her to live a life of celibacy, but would not. She had certainly done her share, and more, with five children contributed to the citizenship of the nation. She with her husband were heroically meeting the bitter dictum of Fate. Their gladnesses had been curtailed sufficiently without tearing away the joys of connubial love. I had this case investigated, and, as the community in which they were supposed to reside was small, I was enabled to be certain that the poor, pitiful plea was a foul lie—a trap. Had I allowed my sympathy and sense of justice to outweigh my prudence, I would doubtless be a convict today. One pathetic appeal pretended to come from the motherless and only daughter of a physician about to marry a young professional man. She did not desire children until they had gained a start; she had read a paper of mine "in a journal in papa's office"; could I tell her, etc. I believed her, but did not tell her. Since then I have had the identical form letter, three times in different sections and in different handwritings. Another told of a dead husband, a brood of children and a pregnancy; could I help her? This case was traced, and found false.

You ask who sets these traps. I do not know, but I am careful to avoid them, and to avoid violation of the law, no matter how unjust and wicked I may consider it. Socialist writers advance the claim that this law has been secured and its enforcement is financed by capitalistic influences; that the overplus of crude labor may glut the markets and render easy the enslavement of the toilers.

Kirkpatrick, in his "War, What For," best expresses what Socialism believes in this line. He says:

"Kings, emperors, czars, capitalists of all lands are interested in the problem of 'race suicide.' Eager that every working-class mother shall become a breeder * * * for more soldiers, for a larger army of the unemployed; that they may, in the industrial civil war, more tyrannically dictate wage terms; more easily secure substitutes."

Mrs. Margaret H. Sanger, the mother of three children, once published a magazine in New York, called "The Woman Rebel," in which she plead for smaller families, such as the worker could adequately rear. She said: "It is only the workers who are ignorant of the knowledge how to prevent bringing children into the world solely to supply the millions of soldiers and sailors to fight the battles, whether for the ruling or the financial classes."

The charitable associations of New York City have conducted a grewsome series of investigations to ascertain the extent of the penury following the enforced idleness caused by the great war. A stevedore was found out of work, with a family of ten children, the oldest thirteen. All were crowded into one horrible hole in a slum tenement. The children were making artificial flowers, down to a little chap of three, and earned 84 cents a day for all. The mother, as was to be expected, was pregnant. Now were I to give to her the simple, wholesome means and knowledge of how to stop this crime of over child-bearing I would be liable to heavier punishment as a criminal than is the average meted for murder.

I use the term "crime of over child-bearing" advisedly, for the bringing of children into the world under such circumstances is a worse crime than the cutting of the throats of the babes at birth. We read of those who "kill the body, and those who slay soul and body."

It is incontestable that the spiritual and intellectual life of children, so born and reared, will be stunted, starved and brutalized; even the mere physical existence will be lowered to the standard of automatons—cheap machines.

A farmer who allows half a dozen or more stalks of corn to grow in a hill will go into bankruptcy. His neighbor who grows but two or three plants in a place, has not only the quality but at the same time a larger aggregate of results, as in the case of the French people of today.

Agriculture emphasizes the necessary thinning and selection for quality as well as production, and then shuts the only scientific avenue to the securing of similar common sense in the most important crop of all, that of humanity.

Mrs. Sanger tells the whole truth in saying that it is the submerged classes—those least able to give development to the citizens of the future—who are most denied the knowledge of control.

The undesirable results follows that those best able to rear children are breeding the smaller families, and the mass of the undeveloped grows proportionately larger all the time, when scientific development of mechanical appliances calls for a diminishing proportion of crude labor.

Under the withering influence of the law as quoted the physician's text-books are forced to silence on this question, and the doctor himself can learn nothing save by indirection. Methods crude, harmful and unscientific are used. The origin of sterilization of the unfit was found in the illegal and criminal operations upon the daughters of wealth who sought an operation and the total loss of maternity as a protection from maternity. When the first vasectomy law was passed in 1907 I hesitated to report it until August, 1908, fearing that the heralding of the new law would be held constructive violation of the Federal law.

Oh, the uncounted thousands of homes that are converted into infernoes, wrecked as a consequence of this gross substitute for decent and harmless methods of prevention.

To be sure, the general diffusion of the knowledge of the prevention of conception would result in some wrongs, the safeguards would be used to allow the gratification of illicit passion, but there is no good that is not capable of perversion, which is sure to be exerted, to a limited extent. No one would close the churches because some hypocrites steal the livery of heaven to serve the devil in.

The decrease of abortion would more than counterbalance the evil.

As the law now stands it is a gross insult to every medical man in the nation. We are classed as a dangerous criminal profession, who must be curbed without recourse. An organized and intelligent effort of the numerous and influential body of American physicians would do much to secure the repeal, or, at least, the amendment of this statute, with all its harshness, injustice, and danger to the Commonwealth.

Doctor, do you know that many of the States have laws even more drastic than that of the United States? Do you know what your own State provides in this respect?

